# Radiological pleuroparenchymal fibroelastosis associated to limited cutaneous systemic sclerosis: a case report

**DOI:** 10.1186/s12890-018-0641-5

**Published:** 2018-05-18

**Authors:** D. Hassoun, S. Dirou, P. P. Arrigoni, C. Durant, M. Hamidou, A. Néel, C. Agard

**Affiliations:** 1 Internal Medicine Department, Hôpital Hôtel-Dieu, Centre Hospitalier Universitaire de Nantes, 1 place Alexis Ricordeau, 44093 Nantes, France; 2 Pneumology Department, Institut du Thorax, Hôpital Guillaume et René Laënnec, Centre Hospitalier Universitaire de Nantes, Nantes, France; 3 Radiology Department, Hôpital Hôtel-Dieu, Centre Hospitalier Universitaire de Nantes, Nantes, France

**Keywords:** Pleuroparenchymal fibroelastosis, Systemic sclerosis, Interstitial lung disease

## Abstract

**Background:**

Pleuroparenchymal fibroelastosis (PPFE) is a very rare interstitial lung disease (ILD) characterized by progressive fibrotic lesions of the visceral pleura and the sub-pleural parenchyma, affecting predominantly the upper lobes. PPFE may occur in different contextes like bone marrow or lung transplantations, but also in the context of telomeropathy with mutations of telomerase reverse transcriptase (*TERT*), telomerase RNA component (*TERC*) or regulator of telomere elongation helicase 1 (*RTEL1)* genes. PPFE-like lesions have recently been described in patients with connective tissue disease (CTD)-related ILD. We report here the first detailed case of PPFE associated to systemic sclerosis (SSc) in a woman free of telomeropathy mutations.

**Case presentation:**

A caucasian 46 year old woman was followed for SSc in a limited form with anti-centromere Ab since 1998, and seen in 2008 for a routine visit. Her SSc was stable, and she had no respiratory signs. Pulmonary function tests showed an isolated decreased cTLCO at 55.9% (of predicted value). Cardiac ultrasonography was normal. Thoracic CT-scan showed upper lobes predominant mild and focal pleural and subpleural thickenings, suggestive of PPFE, with a slight worsening at 8 years of follow-up. She remained clinically stable. Biology only found a moderate and stable peripheral thrombocytopenia, and sequencing analysis did not find any mutations in *TERT* and *TERC* genes.

**Conclusions:**

ILD is frequent in SSc but isolated PPFE has never been described so far. In our case, PPFE is not related to telomeropathy, has indolent outcome and seems to have good prognosis. PPFE might be an extremely rare form of SSc-related ILD, although a fortuitous association remains possible.

## Background

Pleuroparenchymal fibroelastosis (PPFE) is a very rare condition characterized by progressive fibrotic lesions of the visceral pleura and the sub-pleural parenchyma, intra-alveolar and septal structures, affecting mainly the upper lobes [1, 2]. Regarding the 2013 classification of idiopathic interstitial pneumonias, PPFE is considered as a distinct entity among a heterogeneous group of rare interstitial lung diseases (ILD) [1]. Poor is known about this disease that may occur in different conditions such as transplantation or chemotherapy, while idiopathic and familial forms of PPFE are also mentioned. Moreover, PPFE has been reported in family histories of telomeropathy associated to mutations of telomerase reverse transcriptase (*TERT*), telomerase RNA component (*TERC*) or telomere elongation helicase 1 (*RTEL1)* genes [3, 4]. We report here the first case of isolated PPFE in a caucasian patient with systemic sclerosis (SSc) who does not carry *TERT* and *TERC* mutations.

## Case presentation

A caucasian 46 year old woman was seen in 2008 for a follow-up visit. She had a SSc in a limited form since 1998, comprising Raynaud phenomenon, sclerodactily, digital and facial telangiectasia, and sicca syndrome, with anti-centromere antibodies (Abs). She had never developed any other specific organ involvements. She had also thyroid insufficiency since 2005. No familial medical history was noted. Her treatment included ocular cyclosporine, ocular vitamin A, aspirin 75 mg/d, diltiazem 240 mg/d and levothyroxine 75 μg/d. She had never smoked. No toxic occupational exposures were found.

**Fig. 1:**
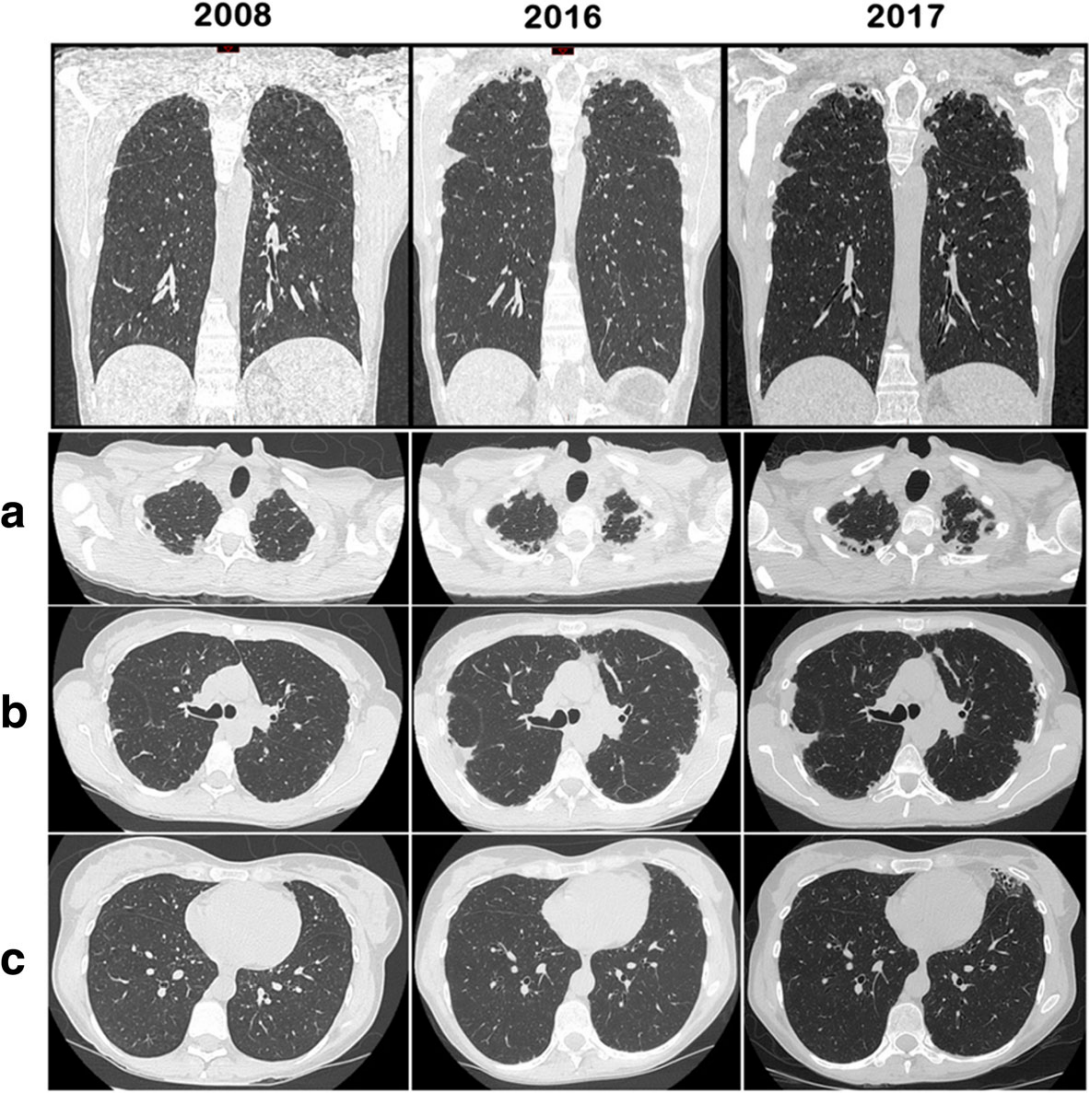
Evolution of thoracic imaging by CT-scan over time. Axial images centered on **a** 2nd thoracic vertebra, **b** 5th thoracic vertebra and **c** 8th thoracic vertebra. Pleural and pulmonary injuries are characterized by fibrotic foci of the upper lobes, without any other signs of SSc-related ILD. An apico-basal gradient of lesions was found. A slow extention of the fibrosis was seen in 2016 and 2017

Weight was stable at 42 kg and body mass index was at 16.4 kg/m^2^ (constitutionnal thinness). Thorax morphology was normal. Skin fibrosis was stable, involving the fingers and the face, with a Rodnan modified skin score (mRSS) at 3. She did not complain of dyspnea or cough and lung auscultation was normal. Usual laboratory tests were normal. Pulmonary function tests (PFT) showed a decreased cTLCO (transfer factor of the lung for carbon monoxide, corrected for hemoglobin) at 55.9% (of predicted value), FVC (forced vital capacity) was at 2.68 L (87.8%) and total lung capacity (TLC) at 5.17 L (108.4%). FEV1/FVC ratio was at 1. Cardiac ultrasonography was normal. Thoracic CT-scan showed upper lobes predominant mild and focal pleural and subpleural thickenings without any other specific lesions (Fig. 1a). She was then followed every year without any signs of active SSc or respiratory signs, and BMI was stable. Treatment was unchanged and PFT remained stable.

In 2016, on the thoracic CT-scan, pleural thickenings appeared more pronounced, associated with bilateral sub-pleural foci of lung fibrosis in both upper lobes (Fig. 1b). At that time, the diagnosis of PPFE was made. 18-Fluorodeoxyglucose (FDG)-PET-scan was performed and found a mild uptake of the pleuro-parenchymal lesions without any suspicion of underlying neoplasia. Various differential diagnosis were ruled out and she did not have any signs suggesting Langerhans Cell Histiocytosis, spondylarthropathy or inflammatory bowel disease. On biologic analysis, a moderate peripheral thrombocytopenia was noted (31-75 G/L) without any other hematologic alterations: hemoglobin at 12 g/dL, leucocytes at 5.64 G/L, including 3.36 G/L neutrophils and 1.13 G/L lymphocytes. Liver tests and renal function (creatinine at 55 μMol/L) were normal. Gammaglobulins were at 9.2 g/L and albumin at 40.5 g/L. Arterial blood gas were normal. Auto-immune profile was stable, with anti-nuclear Abs (1/2560) and anti-centromere Abs, while anti-SSA/Ro, anti-SSB/La, anti-U1RNP Abs and rheumatoid factors were negative. The search for *Mycobacterium tuberculosis* was negative (culture of sputum). Otherwise sequencing analysis did not find any mutations in *TERT* and *TERC* genes. At last evaluation in 2017, patient was stable, there was a slight decrease in cTLCO (48%), FVC (83%) and TLC (88%), while lung involvement was unchanged on CT-scan (Fig. 1c).

## Discussion and conclusions

To the best of our knowledge, this is the first reported case of isolated PPFE associated with SSc in a Caucasian woman. PPFE developed progressively in the decade after initial diagnosis of limited SSc, without any other specific involvement. Imaging showed progressive alterations of lung fibrotic lesions, a slow decrease of TLC and TLCO, with mild uptake of 18-FDG on PET-scan. However, our patient had no dyspnea neither cough or chest pain, and no objective clinical signs suggestive of ILD like basilar crackles. Finally, this case of PPFE has a grumbling pattern outcome.

SSc is a rare connective tissue disease affecting predominantly women, and typically characterized by Raynaud phenomenon, dermal fibrosis, and auto-immunity. Severe organ lesions may occur, like intestinal, renal, myocardic or pulmonary involvements, mainly ILD and pulmonary arterial hypertension that are the two leading causes of mortality in SSc. ILD affects about 80% of SSc patients, but only 15-20% of them, mainly those with diffuse skin involvement and anti-topoisomerase 1 Abs, develop an extensive lung disease of bad prognosis [5]. Most SSc patients with ILD (around 3/4) have a non-specific interstitial pneumonia (NSIP) pattern whereas usual interstitial pneumonia (UIP) remains uncommon [5]. Rare patients may also develop combined pulmonary fibrosis and emphysema syndrome. Our present case raises the question whether PPFE might be considered as a potential additional pattern of ILD associated to SSc.

Recently, radiologic PPFE-like lesions have been described in 21 japanese patients with various connective tissue disease (CTD)-related ILD [6]. In this study, most of these patients had clinical signs of ILD with fine crackles on chest auscultation (17 among 19 patients evaluated). Enomoto et al. found that 6 of 14 patients with SSc-related ILD (43%) had also radiologic PPFE lesions, and this frequency was lower among patients with inflammatory myopathy (11%), rheumatoid arthritis (6%), primary Sjögren’s syndrome (29%) and overlapping CTD (28%). In this original study, lung lesions were commonly progressive and associated to higher risk for respiratory death [6]. Patients with radiologic PPFE-like lesions had also higher frequency of pneumothorax and/or pneumomediastinum than those without (38% vs 15%). Interestingly, mean BMI was significantly lower in patients with PPFE-like lesions (19.8 vs 22.4 in patients without PPFE-like lesions), and this was also the case in our patient who had a BMI at 16.4.

Pathologic proof of PPFE is rarely obtained in CTD patients, and most of diagnosis has been made using CT-scan showing appearance and progression of PPFE-suggestive lesions. For our non-symptomatic patient, we decided to not perform any lung biopsy, and we finally made the diagnosis of “radiologic” PPFE. However, previous pathologic studies have shown that PPFE is typically characterized by upper lobes predominant elastin full fibrotic lesions of visceral pleura and sub-pleural intra-alveolar and septal structures [1, 2, 6, 7]. Pathophysiology of PPFE remains unknown and natural history of the disease usually consists of a progressive extent of fibrotic lesions leading to chronic respiratory failure. There are no specific treatments recommended and lung transplantation might be considered in severe patients. Clinical presentation of PPFE is not specific with predominant exertional dyspnea and chronic cough, but a flat chest and a high susceptibility to pneumothorax have been noted [7]. Around 200 cases of PPFE have been reported worldwide, and some of them occurred during various conditions like bone marrow transplantation, lung transplantation, chemotherapy, radiotherapy, but also immune diseases (spondylarthropathy, hemorrhagic rectocolitis) [7, 8]. PPFE may also be associated to heterozygous mutations in telomere-related genes like *TERT*, *TERC*, or *RTEL1*. In these cases, PPFE affects predominantly females [3, 4]. These telomeropathies are familial genetic disorders due to dysfunction of telomerase enzymes that are meant to reduce the shortening of telomere over time (molecular aging). They might be also associated to idiopathic pulmonary fibrosis, chronic hypersensitivity pneumonitis, but also interstitial pneumonia with autoimmune features, and even SSc-related ILD [3]. Whatever the underlying type of lung fibrosis, these mutations seem to be correlated with a progressive disease [3].

To conclude, our case suggests that PPFE could be specifically associated to SSc, as other known causes of PPFE might be ruled out, especially telomeropathy. Of course, a fortuitous association of SSc and PPFE might also be considered. It is to highlight that PPFE occurred very progressively, in a patient with low BMI and remaining asymptomatic after 10 years of follow-up.

## Abbreviations

ILD: Interstitial lung disease
PPFE: Pleuroparenchymal fibroelastosis
SSc: Systemic sclerosis
